# Supplementary material for: Synthesis of New Triazole-Based Thiosemicarbazone Derivatives as Anti-Alzheimer’s Disease Candidates: Evidence-Based In Vitro Study
Source: Molecules. 2022 Dec 20;28(1):21. doi: 10.3390/molecules28010021 (PMC9821906; doi:10.3390/molecules28010021)
Supplement: Supplementary file 1 [file molecules-28-00021-s001.zip › molecules-2006915-supplementary.pdf]

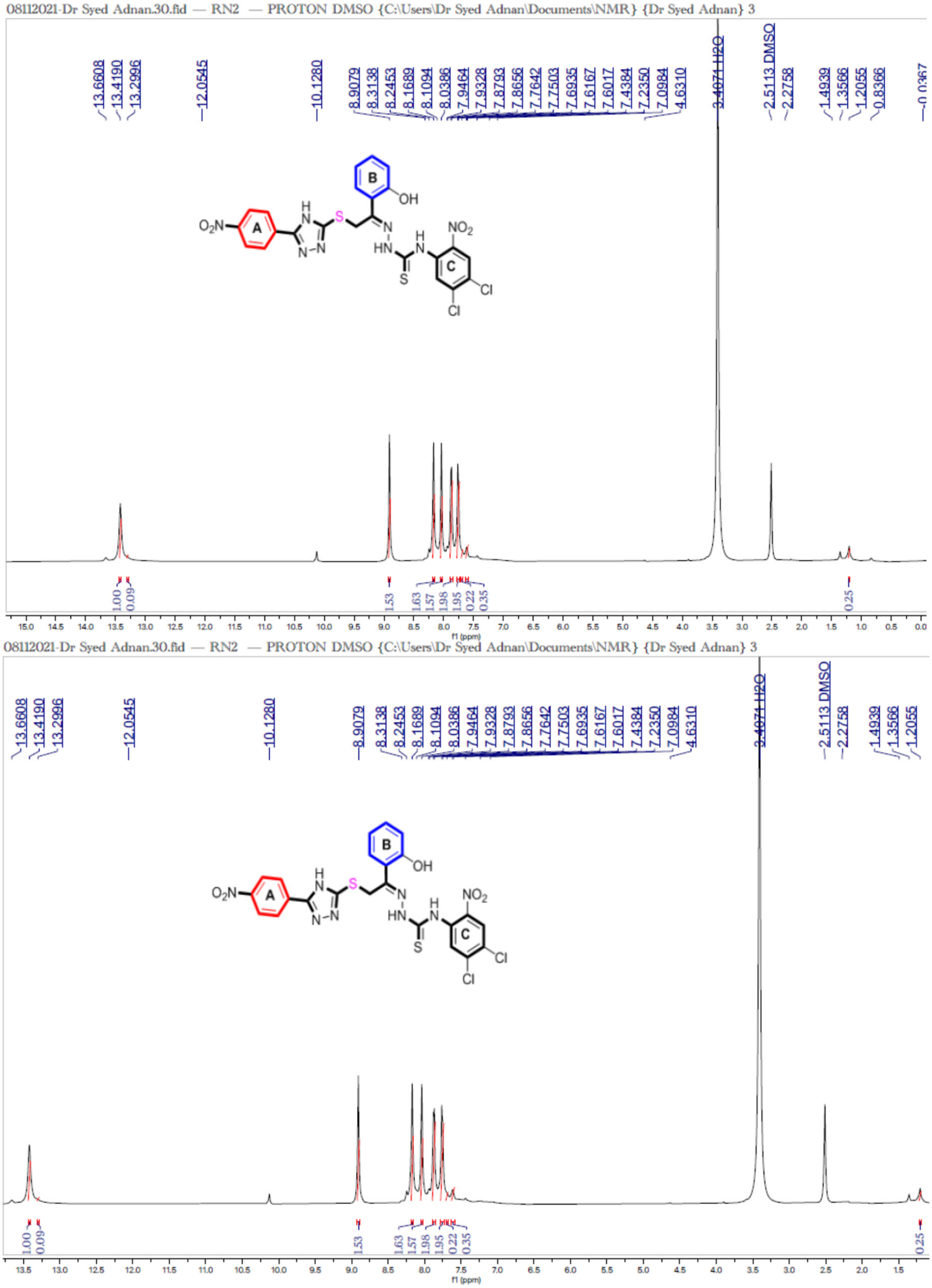


**Figure S1.** Proton NMR spectrum of compound **6b**

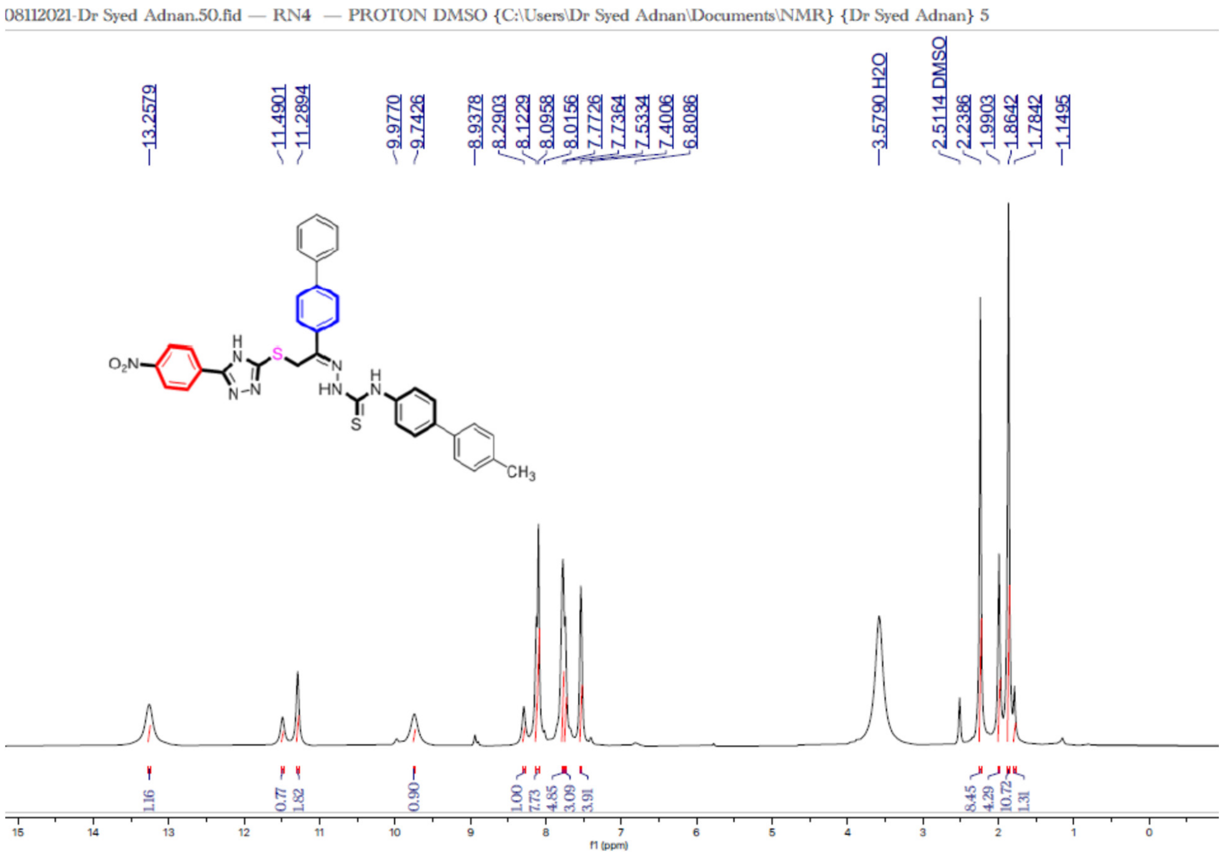


**Figure S2.** Proton NMR spectrum of compound **6d**

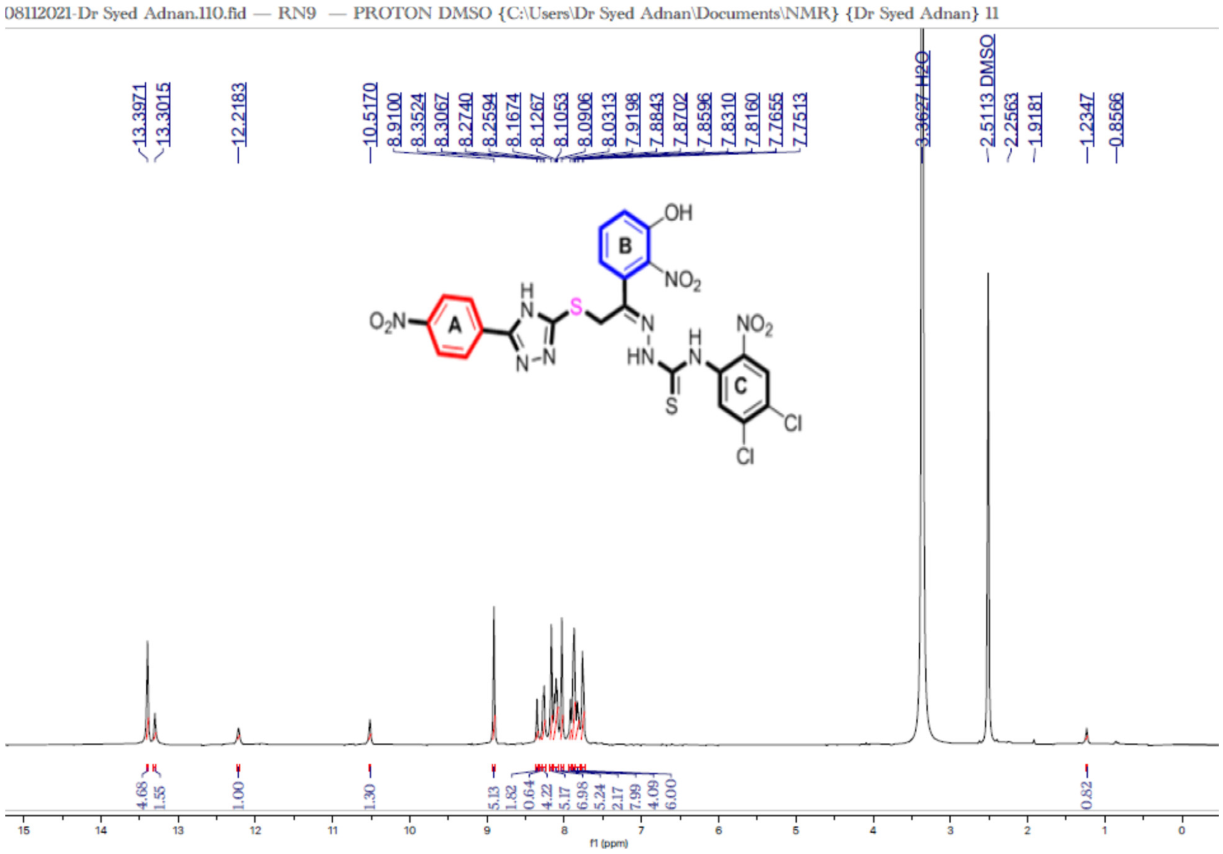


**Figure S3.** Proton NMR spectrum of compound **6i**

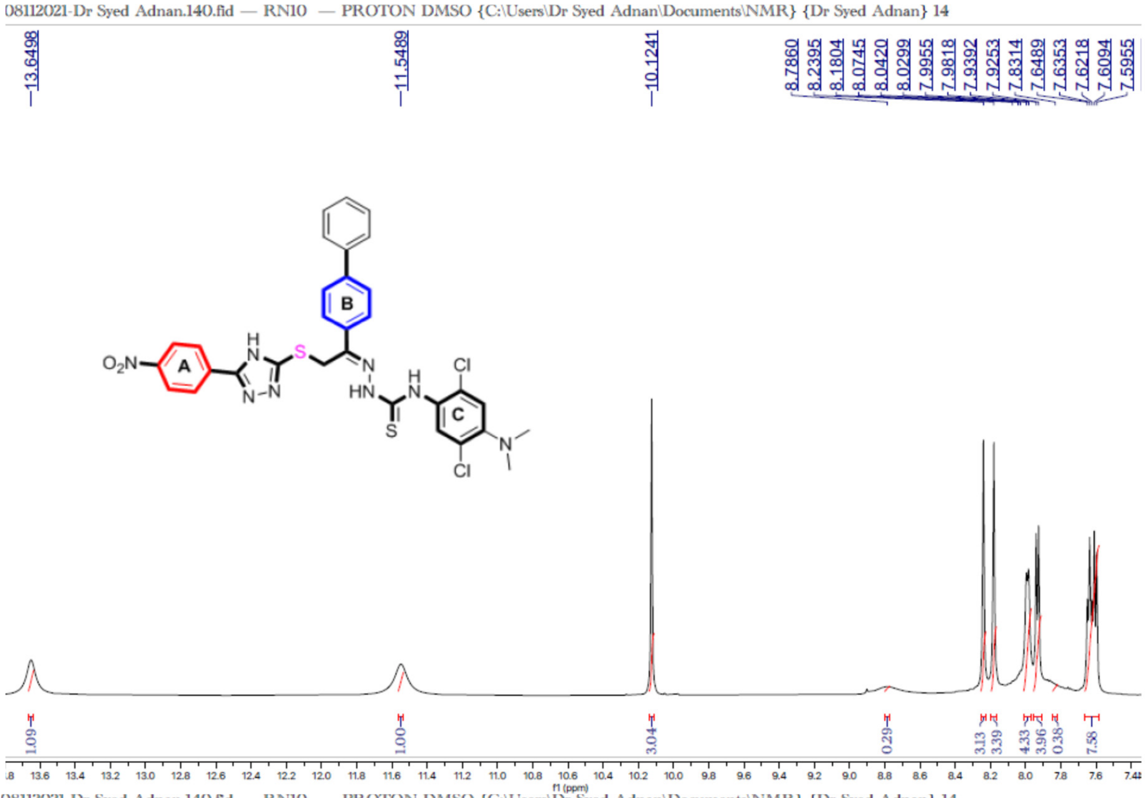


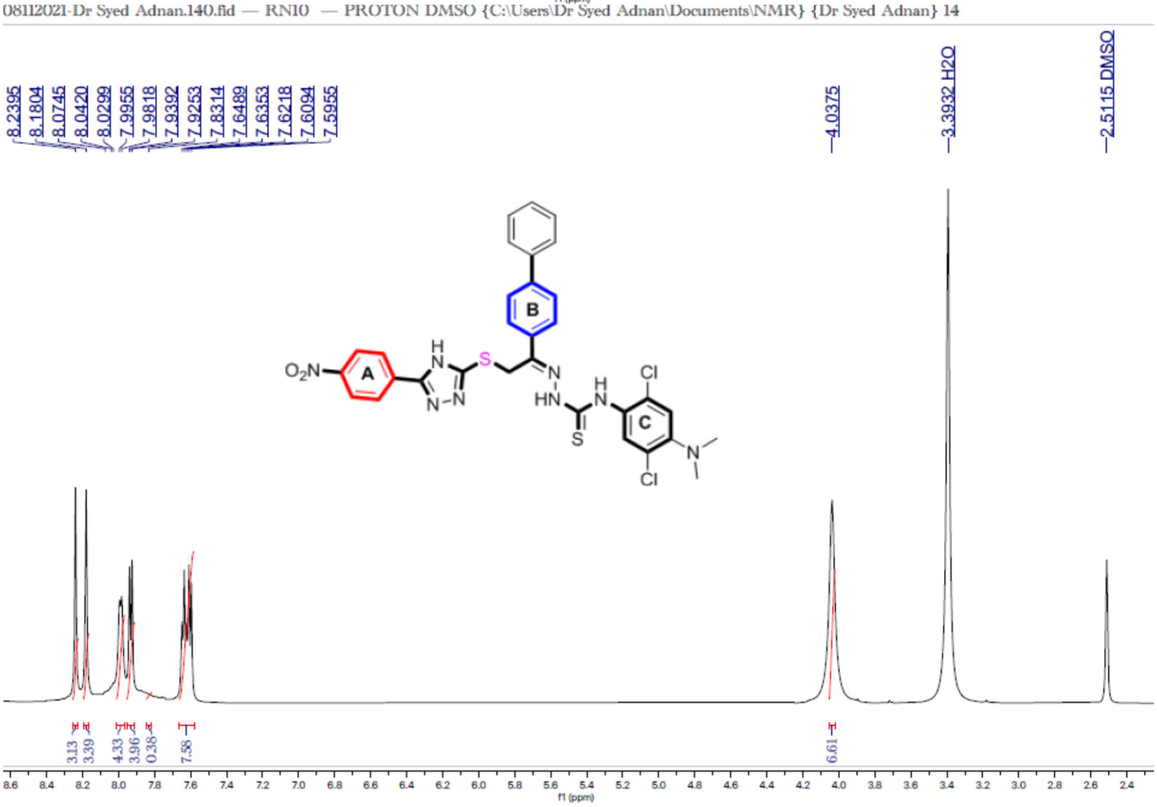


**Figure S4.** Proton NMR spectrum of compound **6j**
